# Toxicity, Cellular Uptake, and Intracellular Fate of Particulate and Dissolved Uranium in Murine Lung-Relevant Cell Models

**DOI:** 10.1021/acsomega.6c06055

**Published:** 2026-09-25

**Authors:** You Cheng Khng, Gianni F. Vettese, Satoshi Utsunomiya, Anna M. Psyrillou, Ole Christian Lind, Per Malmberg, Mirkka Sarparanta, Gareth T. W. Law

**Affiliations:** † Radiochemistry Unit, Department of Chemistry, 3835 The University of Helsinki, Helsinki 00560, Finland; ‡ Singapore Nuclear Research and Safety Institute, National University of Singapore, Singapore 118415, Singapore; § Department of Geosciences, 33561 National Taiwan University, No. 1, Sec. 4, Roosevelt Road, Taipei 106, Taiwan; ∥ Faculty of Environmental Sciences and Natural Resource Management, Norwegian University of Life Sciences, P.O. Box 5003, Ås NO-1432, Norway; ⊥ Department of Chemistry and Chemical, Engineering, 11248 Chalmers University of Technology, Göteborg 412 96, Sweden

## Abstract

Uranium dioxide (UO_2_) particles pose an inhalation hazard in occupational and environmental exposure scenarios, yet their cellular interactions and toxicological behavior remain poorly defined. This study investigated the dissolution behavior and colloid-forming potential of UO_2_ in cell culture media, and compared the cytotoxicity, inflammatory response, and intracellular fate with those from equivalent aqueous uranium (U_(aq)_) exposures in murine RAW264.7 macrophages and Mlg2908 lung fibroblasts *in vitro*. In cell culture media, UO_2_ exhibited low solubility and limited colloid formation (∼5% colloidal), whereas U_(aq)_ showed a higher proportion of colloid species (∼21%). In cellular exposure studies, particulate UO_2_ was generally more toxic than U_(aq)_ across all concentrations (12–120 μg/mL; 50–500 μM) and exposure durations (6–48 h). A significant inflammatory response was observed only in macrophages exposed to 60 μg/mL of UO_2_. Uranium was predominantly recovered in the intracellular fraction of cells exposed to UO_2_ (68–79% of added U), whereas U_(aq)_ exposures remained largely extracellular (∼1% intracellular fraction of added U). TEM showed that UO_2_ particles were localized within membrane-bound vesicular structures. HR-TEM and elemental analyses showed that internalized UO_2_ particles largely retained their chemical integrity after 24 h. Overall, these findings demonstrate that particulate UO_2_ is more toxic than U_(aq)_, and its toxicity *in vitro* reflects cell-type-specific responses and particle stability following internalization, underscoring the importance of U speciation and physico-chemical form in inhalation health risk assessment.

## Graphical abstract

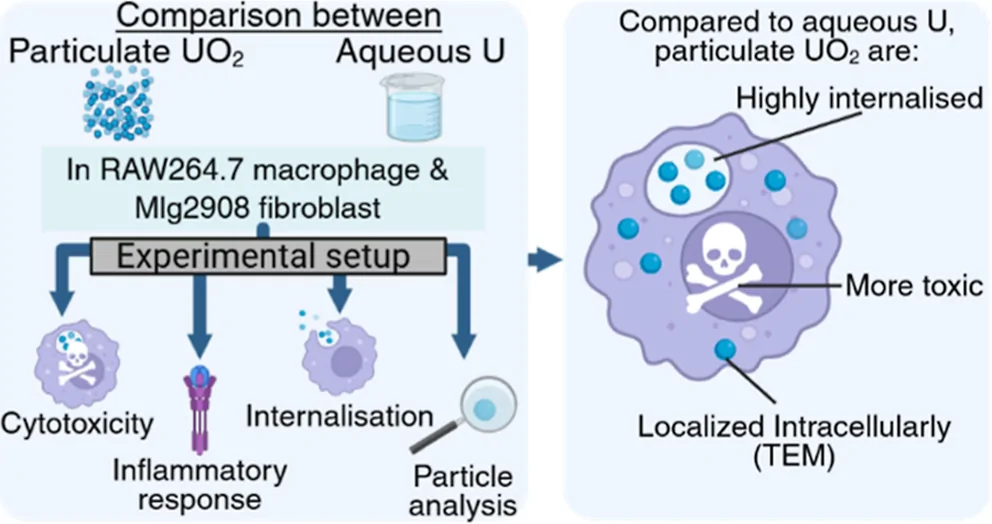

## Introduction

Uranium (U) is a persistent environmental contaminant that poses both chemical and radiological hazards to ecosystems and human health.^1–3^ Its multiple oxidation states strongly influence its mobility and reactivity.^4^ Under reducing conditions, relatively insoluble U­(IV) species dominate, whereas under oxidizing conditions, highly soluble and thus more environmentally mobile, U­(VI) species prevail.^4–6^ U­(V) species are also recognized, though their environmental persistence remains debated.^7–9^ The chemical toxicity of soluble U species (U_(aq)_) is well established, with *in vitro* studies across various cell lines reporting decreased viability, oxidative stress, genotoxicity, and even malignant transformation.^1,3,10,11^ However, these studies predominantly use soluble uranyl­(VI) compounds (e.g. uranyl-acetate, -chloride, or -nitrate), leaving the behavior and toxicity of particulate U forms comparatively poorly understood.

Solid U-bearing particles are also widespread in the environment, yet their behavior and health implications following inhalation are far less understood.^12,13^ U particles arise from diverse sources, including nuclear reactors (accidents, leaks, and waste discharges), industrial processes (mining, milling, and fuel fabrication), and military activities (e.g., nuclear weapons testing and depleted-U munition use).^12–20^ Uranium dioxide (UO_2_) is of particular relevance, as it occurs naturally in reducing environments, constitutes the primary form of mined U ore, is the principal fuel material used in nuclear reactors, and is a corrosion product of spent depleted-U munitions.^21^ Accordingly, UO_2_ particles may become dispersed into the atmosphere during U mining and fuel processing, released as fuel fragments during nuclear accidents, or generated as a corrosion product on spent depleted-U munitions.^17–19,21–23^ Exposure to UO_2_ particles therefore presents both occupational and environmental health concerns, particularly via inhalation.

Particle deposition in the respiratory tract is strongly size-dependent: larger particles (>10 μm) are deposited in the upper airways and readily cleared by mucociliary action, whereas smaller particles (≤10 μm) penetrate into the lower airways, and those ≤5 μm in aerodynamic diameter can reach the alveoli.^24^ There, the absence of cilia precludes mucociliary (e.g., physical) clearance, meaning particles are removed primarily through dissolution into the alveolar lining fluid or phagocytosis by alveolar macrophages. Within the surfactant-rich alveolar environment, U particles may partially dissolve, releasing toxic aqueous U species into interstitial spaces and potentially into the systemic circulation or lymphatic system. Particles that dissolve slowly or form secondary U products may persist for months to years,^25–28^ promoting chronic chemical and radiotoxic effects including inflammation, oxidative stress, and fibrosis.^29,30^ The formation of ultrafine particles (<100 nm) in the alveoli present an additional concern, as they can translocate across epithelial barriers or reach the brain via olfactory pathways.^31,32^

Assuming particles do not dissolve in the alveoli, and without assistance from the cilia, macrophages act as clearance cells that engulf inhaled particles by phagocytosis inside the alveoli.^33,34^ Once internalized, particles are exposed to acidic, enzyme-rich phagolysosomes. Although lysosomal enzymes cannot degrade UO_2_, the acidic conditions of the phagolysosome can promote partial dissolution, as shown in model studies.^27,35^ Dissolution or disaggregation may also produce ultrafine fragments inside the phagolysosome which are capable of escaping into the cytoplasm.^27,36,37^ U-induced disruption of phagolysosomal membranes, likely via interactions with phospholipids and proteins, has also been observed, leading to cytoplasmic release of U species and cell death.^38^ The death of macrophages may hamper clearance, potentially re-releasing partially dissolved or fragmented particles that may then be re-phagocytosed by neighboring cells.^39^ This possible cycle of uptake, lysis, and re-uptake could extend particle residence time and sustain cytotoxicity. Incomplete or “frustrated” phagocytosis is known to drive chronic inflammation.^39^

Although the toxicological evidence base for U is dominated by studies using U_(aq)_, growing evidence suggests that particulate U forms themselves cause equal or greater cellular damage than equivalent dissolved exposures.^11,40^ Enhanced particle toxicity likely stems from particle (and thus toxicant) persistence, poor clearance, and sustained interaction with macrophages and lung epithelial cells. For example, carbon-bearing U particles produced stronger cytotoxic and uptake responses in human lung epithelial cells than U_(aq)_ species, indicating the importance of particle exposure and U speciation.^40^ Likewise, animal studies reveal poor pulmonary clearance of UO_2_, with >90% of particles retained in the lungs for months to years and evidence of U translocation to lymph nodes and other organs.^25,26^ Such long-term retention increases cumulative chemical and radiological dose, elevating the risk of fibrosis and tumor formation.^41,42^

The above findings underscore that U particle toxicity cannot be inferred solely from studies using U_(aq)_ compounds. Indeed, the toxicity, dissolution, and cellular fate of particulate U depend strongly on mineralogy, oxidation state, surface chemistry, and particulate size. For example, UO_2_, U_3_O_8_, and UO_3_ display markedly different dissolution behaviors,^43^ and re-crystallization of secondary U species in lung environments is only beginning to be investigated.^27^ Consequently, extrapolating biological responses from one U phase to another, or equating them to U_(aq)_, risks serious misrepresentation of exposure hazards. To address these uncertainties, this study examines the reactivity, solubility, and cellular responses to UO_2_ particles in murine cell models representative of the human lung. RAW264.7 macrophages (widely used surrogates for alveolar macrophages) and Mlg2908 lung fibroblasts were selected to represent phagocytic and non-phagocytic cell types. These cell lines were chosen because macrophages act as the first line of defense against inhaled particles, whereas fibroblasts reflect structural lung cells responding to secondary inflammatory and oxidative stress. This comparative design enables assessment of both phagocytosis-dependent and independent toxicity mechanisms.

## Results and Discussion

### UO_2_ Dissolution and Particle Behavior in Cell Media

We first studied the dissolution behavior of UO_2_ and assessed the potential formation of U-containing colloids in cell culture media. Secondary aqueous or colloidal U species may exhibit mobility and cellular interactions different from those of UO_2_ particles, thereby potentially confounding interpretations of cytotoxicity results if not adequately addressed.^44,45^ These experiments were conducted in complete cell culture media for RAW264.7, which has a similar chemical composition to that used for Mlg2908 (Table S1).

Ultrafiltration analysis of suspensions containing 120 μg/mL UO_2_ incubated in complete cell culture media for 24 h showed that 76.2% of the U remained in the particulate fraction (>100 nm), suggesting partial dissolution of the particles (Figure 1). As for the dissolved fraction, the majority was present as low-molecular-mass (LMM) species (<1.5 nm), accounting for 18.9% of total added U. The remaining fractions of small (1.5–100 nm) and large (>100 nm) colloids were detected at 4.1% and 0.8% of total added U, respectively. In contrast, PhreeqC modelling (Supporting Information, Section S2) predicted complete dissolution of UO_2_ under these conditions, with no indication of secondary U phase precipitation (Supporting Information S2.2), noting that thermodynamic modelling cannot account for mechanical particle breakdown or fragmentation to colloids. The dominant aqueous U species were predicted to be carbonate complexes, primarily UO_2_(CO_3_)_4_^4–^ and mixed Ca/Mg-uranyl carbonate species, consistent with the high carbonate content of culture media.^46^ The predicted speciation remains informative for assessing secondary U phase formation, indicating no thermodynamic driving forces for secondary U precipitation. However, it is crucial to note that PhreeqC modelling assumes equilibrium and neglects kinetic constraints, particularly over the short time scale considered here, whereas UO_2_ dissolution could be long-term if particles are retained in the lungs. However, we note that past long-term studies (days to months) in simulated lung fluids have also shown that UO_2_ either remains sparingly soluble, or has low apparent solubility due to secondary phase formation.^27,28,43^

**Figure 1:**
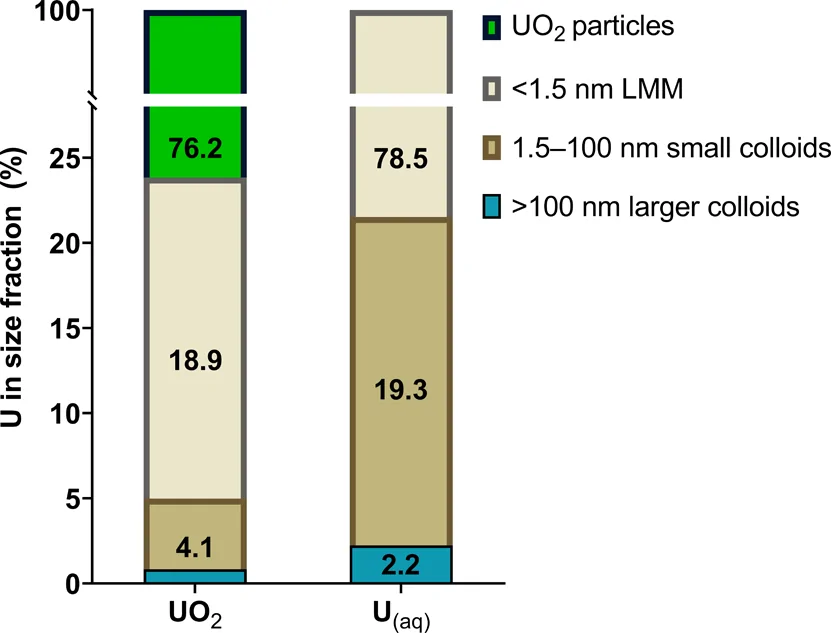
Ultrafiltration results of U size fractions after reacting 120 μg/mL of UO_2_ and aqueous U_(aq)_ with cell culture media for 24 h. Green, grey, brown and blue represents UO_2_ particles, <1.5 nm LMM, 1.5–100 nm and >100 nm colloids, respectively.

We similarly assessed the speciation and size distribution of 120 μg/mL U_(aq)_ (corresponding to ∼50–500 μM total U) in RAW264.7 media for 24 h. Most of the U also remained in the LMM fraction (78.5%) but there was a substantially larger proportion of colloidal U as compared to the UO_2_ systems, with 19.3% and 2.2% present as small and large colloids (≥1.5 nm), respectively (Figure 1). Dynamic light scattering (DLS) analysis was subsequently performed on the <0.1 μm fractions of both UO_2_ and U_(aq)_ suspensions. However, direct characterization was not feasible as the detected size distributions closely resembled those of the media-only control, and no U-specific colloidal population could be resolved above the media-derived background colloids (Figure S3).

Protein corona formation on the UO_2_ particles was assessed by zeta-potential measurement. Particles incubated in complete culture medium for 24 h and washed into TRIS buffer showed a less negative zeta potential than naked particles suspended in the same buffer (−14.0 ± 1.3 versus −24.6 ± 3.3 mV), consistent with adsorption of serum proteins onto the particle surface. The corona-bearing particles nonetheless retained a moderately negative surface charge, consistent with limited particle stability and a propensity for partial aggregation under the exposure conditions. PhreeqC modelling also predicted the lack of secondary U phase precipitation under such conditions (Supporting Information S2.3). Taken together, these results indicate that although partial dissolution of UO_2_ occurs in cell culture media, colloidal U represented a small fraction (∼5%) of total U in the UO_2_ system, whereas a substantially larger colloidal fraction (∼20%) was present in the U_(aq)_ system.

### Cell Viability and Inflammatory Response

To evaluate the cytotoxic effects of particulate and soluble form of U, we quantified cell viability in RAW264.7 macrophages and Mlg2908 fibroblasts following exposure to UO_2_ particles or U_(aq)_. Cell viability was evaluated by measuring total adenosine triphosphate (ATP) levels using a CellTiter-Glo assay after 6, 24, and 48 h of exposure across a concentration range of 12–120 μg/mL UO_2_. This concentration range (12–120 μg/mL UO_2_, ∼50–500 μM total U; see Supporting Information S5) was selected to correspond with ranges used in comparable in vitro studies of environmentally and occupationally relevant uranium exposure in lung cell models. e.g.^10,40,47–49^ The UO_2_ particles were prepared in absolute ethanol to ensure sterility. After diluting with cell culture media, the final ethanol concentration was <0.1% (v/v) and is not expected to influence cell viability.

Overall, exposure to particulate UO_2_ had a greater impact on cell viability than exposure to U_(aq)_ at equivalent concentrations across both cell lines and most time points (Figure 2). This is consistent with previous findings that particulate U is more toxic than U_(aq)_ at equivalent concentrations in A549 human epithelial cells.^40^ A clear dose-dependent toxicity was also observed in both systems, indicating that the selected exposure range was suitable for resolving toxicity effects, and toxicity also increased with exposure duration. In RAW264.7 macrophages, there was no statistically significant reduction in cell viability at the 6 h time point except for 120 μg/mL UO_2_ (Figure 2A), whereas significant toxicity was observed at 24 and 48 h (Figure 2B,C). In contrast, Mlg2908 fibroblasts exhibited reduced viability at earlier time points, indicating greater initial sensitivity to UO_2_ exposure (Figure 2D). At 24 h, both cell lines displayed comparable toxicity profiles (Figure 2E). However, by 48 h, RAW264.7 showed greater loss of cell viability than Mlg2908, suggesting a higher tolerance to prolonged UO_2_ exposure in the fibroblasts (Figure 2F). Moreover, at 48 h, Mlg2908 fibroblasts exposed to U_(aq)_ did not display a clear dose-dependent toxicity. This behavior is likely attributable to near-confluent cell densities at this point, which may result in nutrient depletion, changes in cellular behavior, or signal saturation, leading to inaccurate measurements.^50^ More importantly, this deviation does not alter the general observation that UO_2_ exposure resulted in more pronounced cytotoxicity than U_(aq)_ for both cell types. Moreover, similar variability in dose–dependent toxicity behavior has been reported for U particulates in lung epithelial cell models, where cytotoxicity depended strongly on both cell type and U oxidation state (UO_3_ vs U_3_O_8_) rather than mass concentration alone.^51^

**Figure 2:**
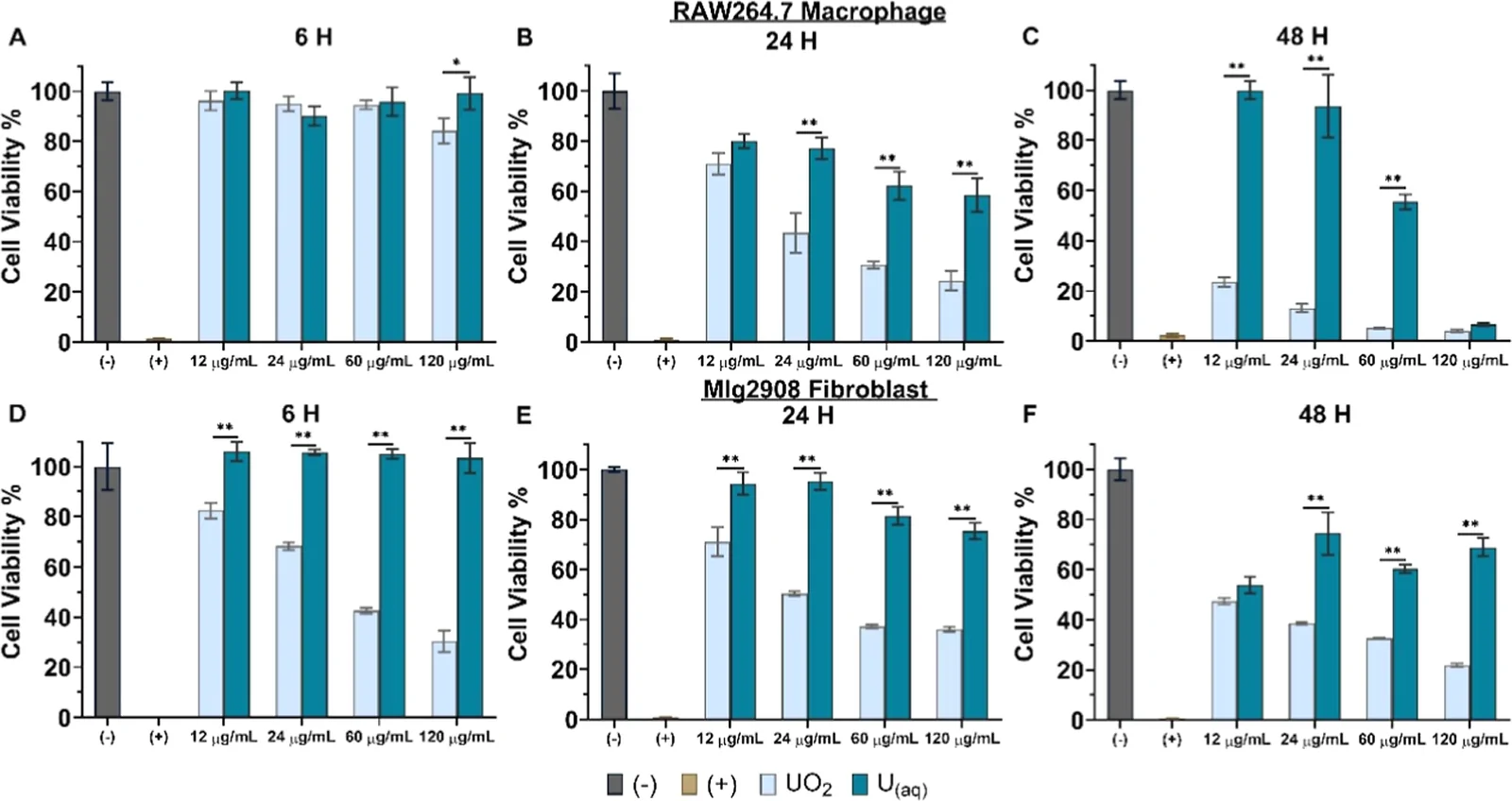
Cell cytotoxicity studies of cell lines following exposure to cell culture media (negative control), 1% triton-X (positive control), and UO_2_ and aqueous U_(aq)_ at concentrations of 12, 24, 60, and 120 μg/mL. Data is reported for RAW 264.7 macrophage cell viability after (A) 6, (B) 24, and (C) 48 h exposure, and Mlg2908 fibroblast cell viability after (D) 6, (E) 24 and (F) 48 h exposure. The cell viability % was obtained by normalizing against the negative control. Data are from triplicates (*n* = 3). The statistical significance was analyzed using one-way ANOVA and post-hoc Tukey test, where the significance was set at *p < 0.05 and **p < 0.01.

To investigate potential mechanisms of cell death due to U exposure, we assessed the inflammatory response by measuring the secretion of TNF-α, a pro-inflammatory cytokine commonly used as a biomarker of immune activation.^52^ TNF-α is a key mediator involved in the initiation and amplification of inflammatory responses, and is secreted by most immune cells, including macrophages.^52^ RAW264.7 macrophages and Mlg2908 fibroblasts were exposed to UO_2_ and U_(aq)_ at two different concentrations (24 and 60 μg/mL) for 24 h. Here, the lowest concentration (12 μg/mL) was excluded because it was not expected to induce detectable levels of TNF-α, while the highest concentration (120 μg/mL) was omitted due to excessive toxicity (Figure 2B,D). The data were normalized against the total protein concentration in each corresponding cell lysate to account for cell death. Complete cell culture media without additives served as the negative control, while 10 ng/mL LPS was used as positive control as it is known to induce secretion of TNF-α in macrophages.^53^ In RAW264.7 macrophages, exposure to 60 μg/mL UO_2_ resulted in a significant increase (*p* < 0.01) of TNF-α secretion relative to the negative control (Figure 3). No significant TNF-α production was observed following exposure to U_(aq)_ at either concentration. These results suggest that the pro-inflammatory response can be attributed to the higher toxicity effect of particulate U at elevated concentrations. As expected, LPS exposure produced a robust TNF-α response, serving as a positive control. In Mlg2908 fibroblasts, TNF-α levels remained below the detection limit for all treatments, consistent with their inherently low cytokine production capacity.^52^

**Figure 3:**
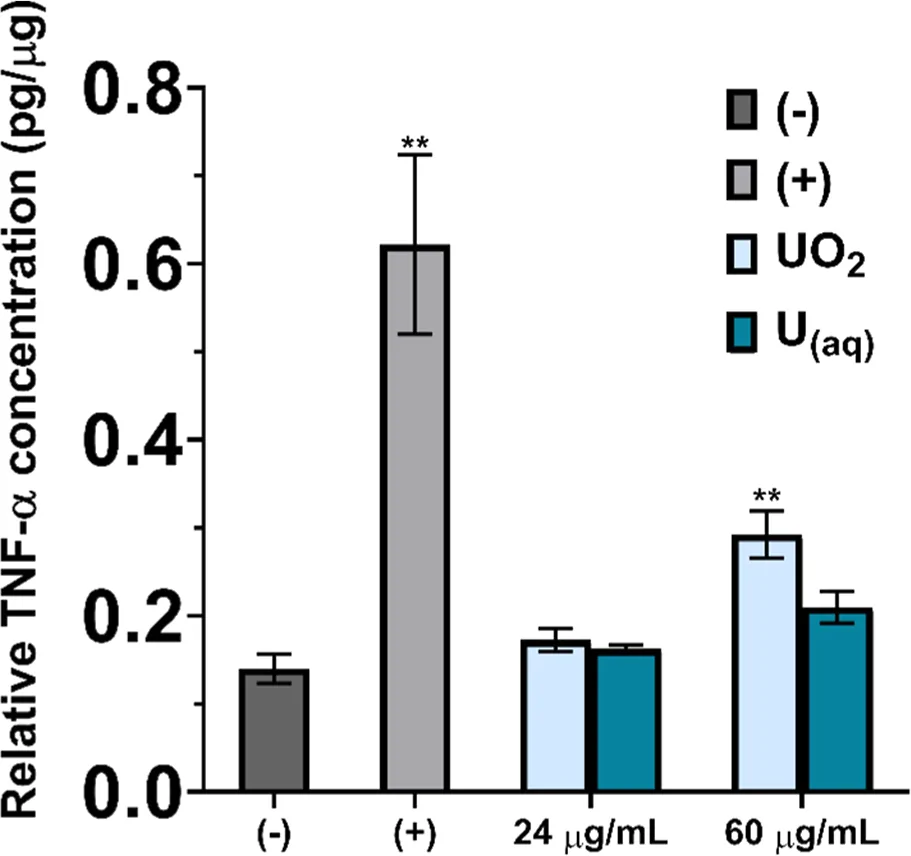
TNF-α protein secretion in RAW264.7 macrophage cells after 24 h exposure to cell culture media (negative control), 10 ng/mL LPS (positive control), and 24, and 60 μg/mL of UO_2_ or U_(aq)_, measured using ELISA kit. Data are from triplicates (*n* = 3). The statistical significance was analyzed using one-way ANOVA and post-hoc Tukey test compared to the control, where the significance was set at *p < 0.05, **p < 0.01. Data for Mlg2908 is omitted because no significant levels of TNF-α were measured.

### Intracellular Uptake Studies

Since particle uptake plays a critical role in determining cell cytotoxicity, we investigated cell internalization of U in RAW264.7 macrophage and Mlg2908 fibroblast following exposure to particulate and U_(aq)_. To minimize UO_2_-induced cytotoxicity cell death that may affect the observation of particle uptake, we used the lowest concentration of 12 μg/mL (50 μM total U).

We first performed intracellular uptake to quantify the localization of UO_2_ and U_(aq)_ within the cells. Cells were exposed to UO_2_ or U_(aq)_ for 6 and 24 h, after which U associated with three defined fractions were quantified: (i) the free fraction (culture supernatant and initial washes), (ii) membrane-bound (acid-washed surface-associated U), and (iii) internalized fraction (intracellular U following cell lysis). U concentrations in each fraction were determined by ICP–MS. Only a small fraction of U was internalized in both cell lines following U_(aq)_ exposure (1.5 ± 0.5% of total U for RAW264.7 and 0.6 ± 0.1% of total U for Mlg2908), indicating minimal cellular uptake of U_(aq)_ under these conditions (Figure 4). No measurable U was detected in the membrane-bound fraction. In contrast, exposure to particulate UO_2_ resulted in substantial cellular uptake in both RAW264.7 macrophages and Mlg2908 fibroblasts. Across both cell lines and time points, 68–79% of the total added particulate U was recovered in the internalized fraction. Less than 5% was detected in the membrane-bound fraction, indicating that most cellular associated U originated from internalization rather than surface adsorption. Comparable levels of internalized U observed after 6 and 24 h exposure suggest that uptake of UO_2_ particles occurred predominantly within the first 6 h. While RAW264.7 macrophages showed substantial intracellular U accumulation at 6 h without an accompanying reduction in viability, Mlg2908 fibroblasts exhibited both rapid uptake and an immediate decrease in viability over the same exposure time (Figure 2). This rapid internalization consists of particle-driven uptake mechanisms such as endocytosis or phagocytosis and indicates that prolonged exposure primarily increases intracellular residence time rather than uptake efficiency.^40^ These results demonstrate a marked contrast between particulate and dissolved U with respect to cellular association. While U_(aq)_ remained largely extracellular, UO_2_ particles were efficiently internalized by both phagocytic and non-phagocytic cells, resulting in high intracellular U burdens. This differential uptake behavior provides a mechanistic basis for the enhanced cytotoxicity observed for UO_2_ relative to U_(aq)_ and motivates further investigation of the intracellular fate of internalized particles.

**Figure 4:**
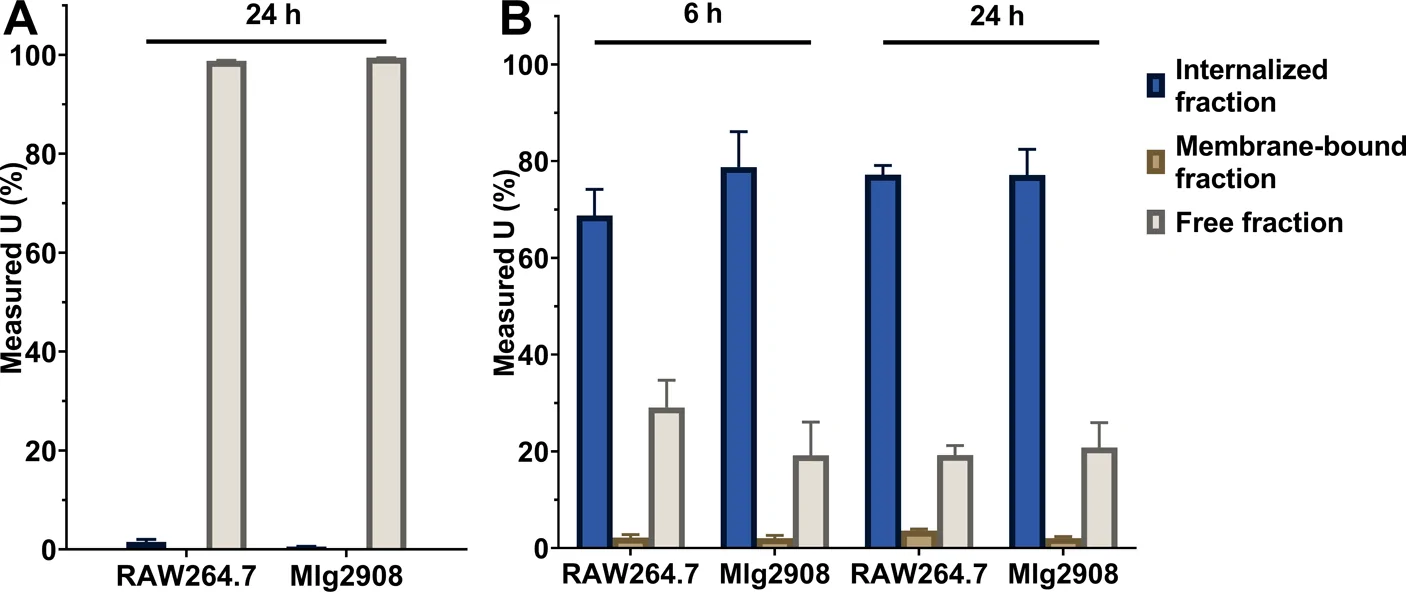
Intracellular accumulation of U in RAW264.7 macrophages and Mlg2908 fibroblasts after (A) 24 h exposure to 12 μg/mL of U_(aq)_, and (B) 6 and 24 h exposure to 12 μg/mL of UO_2_. Error bars represent one standard deviation of internalized U from replicate (*n* = 3) experiments. Error bars are within symbol size if not visible.

### Intracellular Localization and Fate of Internalized UO_2_ Particles

To further investigate the intracellular fate of internalized U particles, we performed transmission electron microscopy (TEM) on ultrathin (∼60 nm) cross sections of exposed cells. Figure 5 shows the TEM images of RAW264.7 macrophages and Mlg2908 fibroblasts exposed to 12 μg/mL (50 μM total U) of UO_2_ (Figure 5A–C,E–G) or U_(aq)_ for 24 h (Figure 5D,H).

**Figure 5:**
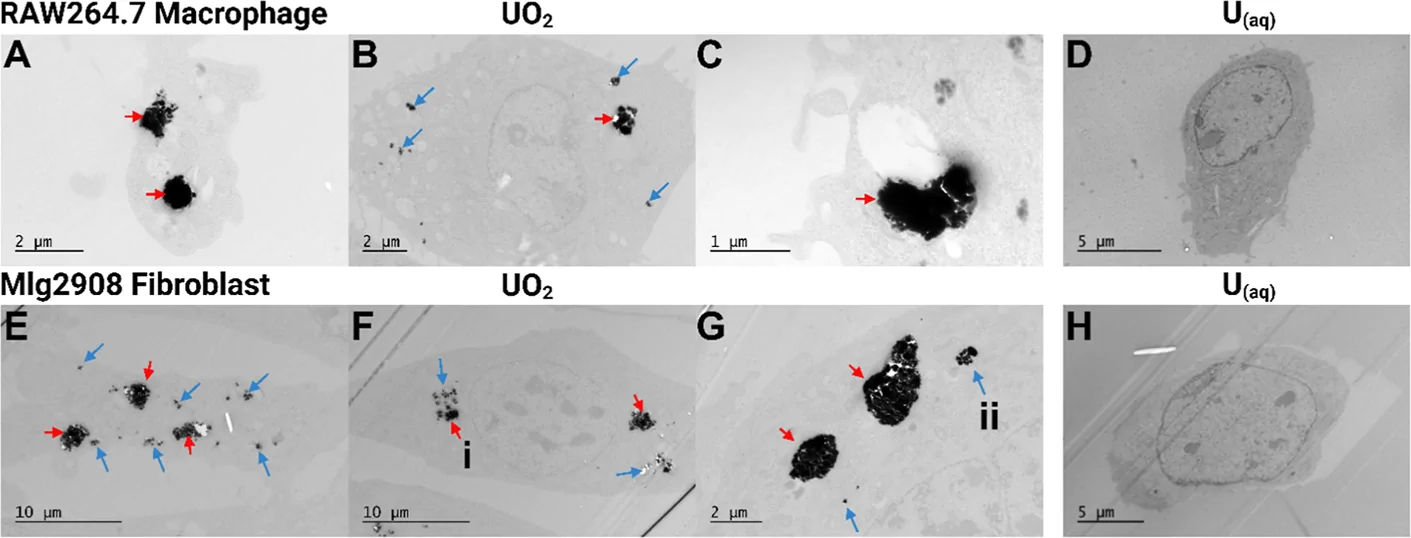
TEM images of ultrathin sections (∼60 nm) of RAW264.7 macrophages exposed to 12 μg/mL of (A–C) UO_2_ and (D) U_(aq)_ for 24 h, and of Mlg2908 fibroblasts exposed to 12 μg/mL of (E–G) UO_2_ and (H) U_(aq)_ for 24 h. Red arrows indicate μm-sized particles while blue arrows indicate nano-sized particles. Labels i and ii indicate particles localized within the cytoplasm.

TEM imaging revealed the presence of electron-dense particles within the cytoplasm of both cell lines following exposure to UO_2_ (Figure 5A–C,E–G). These particles were absent in untreated controls and cells exposed to U_(aq)_, indicating that intracellular electron-dense features originated from internalized UO_2_ rather than from intracellular precipitation or background artefacts (Figure 5D,H). Larger μm-sized particles were predominantly localized inside vesicular structures (indicated by red arrows in Figure 5A–C,F,G), suggesting that the mechanism of particle uptake is likely endocytosis in both macrophages and fibroblast cells. Similar vesicular internalization of UO_2_ has also been reported in alveolar macrophages within hours of exposure, where particles were predominantly confined in phagolysosomal compartments.^38^ Additionally, nano-sized particles were also observed within the cytoplasm and outside of vesicular structures (blue arrows). They were likely internalized followed by endosomal escape.^54^ Some apparently larger particles also appear to be within the cytoplasm (Figure 5i,ii), which may be due to nano-size particles clustering extracellularly prior to particle uptake. Similar observations were also reported in a study using carbon-bearing nanosized particles.^40^ Cells exposed to U_(aq)_ do not have any observable electron-dense dark spots (Figure 5D,H), thereby demonstrating that (i) colloid formation does not likely contribute to U uptake, and (ii) U does not appear to precipitate inside both cell lines, likely as it is not saturated within the cells. In contrast, Huang et al. (2022) reported that both extracellular and intracellular uranyl precipitates were observed at higher U_(aq)_ concentrations (>300 μM or 71 μg/mL in non-renal cells).^55^ Together, these observations suggest that UO_2_ may be toxic even at low concentrations (12 μg/mL) due to direct particle uptake, while U_(aq)_ toxicity at higher concentrations may be associated with subsequent precipitation.

To further investigate the fate of internalized UO_2_, HAADF-STEM coupled with EDX and HRTEM were performed on RAW264.7 macrophage exposed to 12 μg/mL of UO_2_. HAADF-STEM imaging confirmed the presence of nanoscale UO_2_ particles within the cytoplasm (Figure 6A) that were observed in Figure 5 (blue arrows). At higher magnification, the particle retained a largely intact structure (Figure 6B). Adjacent smaller particles were also observed, which may reflect minor surface disruption or presence of separate nanoscale particles, although these could not be easily distinguished. EDX elemental mapping identified U as the dominant constituent, with no detectable enrichment of P or Ca indicative of secondary U phases under these conditions and time scales (Figure 6B,C). To assess potential chemical heterogeneity within the particle, EDX spectra were collected from three distinct regions: an adjacent nano-sized (∼40 nm) particle (Figure 6C­(i)), the surface of the main UO_2_ particle (Figure 6C­(ii)), and its core (Figure 6C­(iii)). All regions exhibited similar elemental composition containing mainly U, suggesting the absence of detectable chemical alteration. Consistent with this, HR-TEM images (Figure 6D) revealed little evidence of structural degradation or surface transformation. Analysis of other similar particles are provided in Figure S1. Taken together, the nano-sized UO_2_ particles remained chemically and structurally stable in the cytoplasm after 24 h. However, the relatively short timeframe may be insufficient for significant chemical alteration or reaction of UO_2_ to an extent detectable by the techniques employed. This behavior contrasts with longer-term or more chemically sensitive experimental approaches reported previously, where surface alteration of UO_2_ was observed after extended exposure in simulated lung fluids, highlighting the importance of time scale when interpreting particle transformation processes.^27^

**Figure 6:**
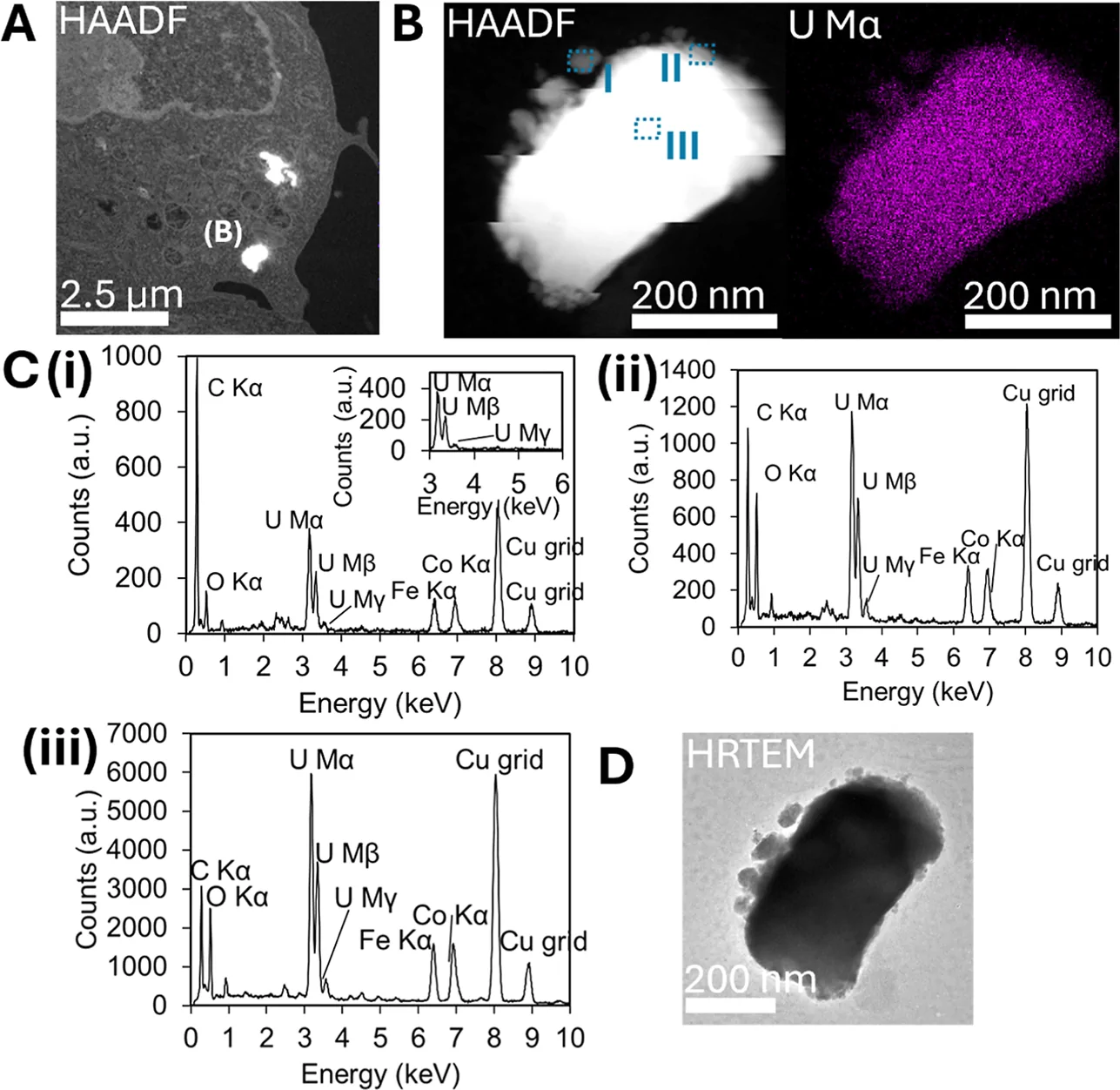
HAADF–STEM analysis of an ultrathin section (∼60 nm) of RAW264.7 reacted to 12 μg/mL of UO_2_ for 24 h. (A) HAADF image of UO_2_ particles inside the cytoplasm. The particle of interest is denoted as (B). (B) Zoomed in HAADF image and EDX mapping of U (purple) of UO_2_ particle marked in (A). (C) STEM–EDX spectrum of regions as labelled in (B). Region I refers to an ultrafine UO_2_ particle (∼25 nm), region II refers to the surface of the UO_2_ particle, and region III refers to the core of the particle. (D) HR-TEM image of the particle of a zoomed in image on the ultrafine particle.

Figure 7 shows TEM data from Mlg2908 fibroblast cells exposed to 12 μg/mL UO_2_. μm-sized internalized UO_2_ particles were observed, with possible indications of slight disruption (Figure 7A). EDX elemental mapping in Figure 7B showed that the structure comprised mainly U, with no detectable P or Ca, indicating minimal chemical alteration. EDX spectrum (Figure 7C) exhibited a similar elemental composition as observed in the RAW264.7 macrophages (Figure 6C), further suggesting that the UO_2_ particle remains chemically unaltered despite having structural disruption. SAED analysis in Figure 7D exhibited concentric diffraction rings characteristic of a polycrystalline UO_2_ structure, with discrete bright spots. These patterns suggest that the UO_2_ particles retained a polycrystalline structure with partial preferential crystal orientation, consistent with physical alteration rather than chemical transformation. HR-TEM analysis (Figure 7E) showed signs of particle fragmentation, TEM analysis of other similar particles are provided in Figure S2. Interestingly, this was not observed in the macrophage uptake studies (Figures 6D and S1). However, with our current data set, it is difficult to unambiguously determine whether UO_2_ fragmentation only occurs in fibroblast cells. Further work is required to clarify the underlying cause.

**Figure 7:**
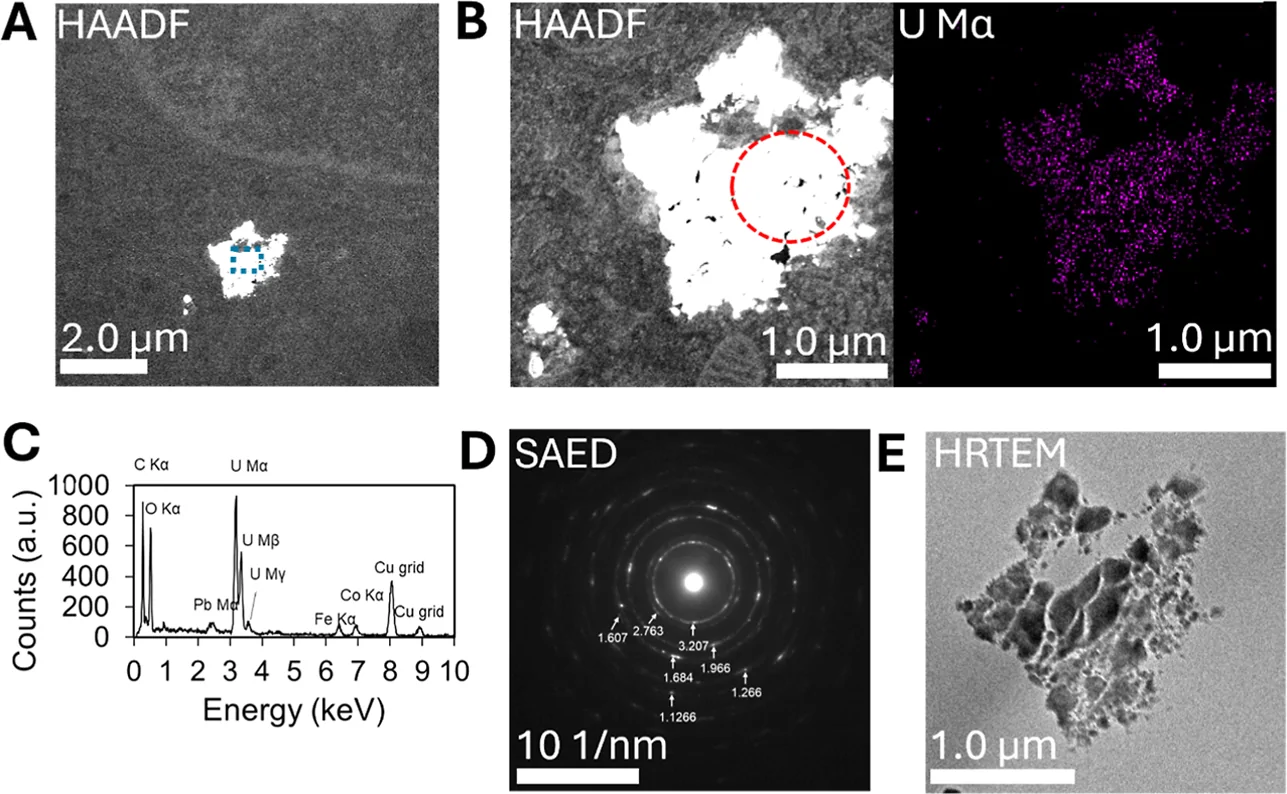
HAADF–STEM analysis of an ultrathin section (∼60 nm) of Mlg2908 reacted to 12 μg/mL of UO_2_ for 24 h. (A) HAADF image of a UO_2_ particle inside the cytoplasm. (B) Zoomed in HAADF image and EDX mapping of U­(purple) of UO_2_ particle marked in (A). The red circle refers to regions where SAED was obtained (C) STEM–EDX spectrum of regions as labelled in (A). (D) SAED patterns from areas highlighted in (B). (E) HR-TEM image of the particle of a zoomed in image on the ultrafine particle.

TEM imaging showed that many larger UO_2_ particles were located within membrane-bound vesicular structures, consistent with uptake by endocytic/phagocytic pathways, while smaller electron-dense particles were also observed intracellularly. Detailed HAADF–STEM, STEM–EDX, SAED, and HR-TEM analyses were performed on selected intracellular particles that were suitable for high-resolution imaging and elemental mapping in ultrathin sections. These analyses showed that the selected particles remained U-rich and retained UO_2_-like structural features after 24 h exposure. However, the analyzed particles should not be considered representative of all intracellular UO_2_, and the data do not provide a compartment-specific assessment of particles within lysosomal/phagolysosomal vesicles. Therefore, the findings that UO_2_ retained its chemical and structural integrity is limited to the particles analyzed here; further compartment-specific studies would be required to determine whether vesicular or lysosomal particles undergo different alteration or dissolution behavior.

### Implications

Overall, these findings provide important insights into the behavior and toxicity of UO_2_ particles in cellular models and their relevance to inhalation exposure. Here, UO_2_ particles dissolved more readily in complete cell culture media than under physiologically relevant simulated lung fluid (SLF) conditions, consistent with laboratory UO_2_ dissolution studies^27^ and reports for other metal oxides.^56,57^ This difference likely reflects the higher abundance of complexing ligands and anions in culture media necessary for cell growth, which can enhance U dissolution. This may obscure particle dissolution chemistry under inhalation-relevant conditions, where ligand concentrations are reportedly lower.^57^

Some degree of UO_2_ particle agglomeration and protein corona formation may also be expected in complete cell culture media containing 10% FBS. As the UO_2_ starting material is produced by sintering and crushing, it already comprises agglomerated polycrystalline grains, and our data cannot unambiguously distinguish whether internalized particles were predominantly taken up as fine fractions that subsequently agglomerated, as larger agglomerates that broke down intracellularly, or with their size unchanged. The UO_2_ exposure should therefore be considered a heterogeneous population of individual particles and agglomerates with associated protein coronas, rather than a monodisperse suspension. These properties may influence particle sedimentation, cellular interaction, and uptake, and the lack of full characterization such as BET analysis in biological media limits quantitative correlation between administered and delivered particle dose. Further studies characterizing UO_2_ agglomeration, breakdown, and protein corona formation in biological media are warranted. Moreover, while cell culture systems enable direct investigation of particle–cell interactions and intracellular processes, they are generally constrained by short exposure durations (<48 h) and are therefore more suited to probing acute cellular responses. Particle residence time in the lungs cannot be determined from *in vitro* studies alone, and *in vivo* investigations are therefore necessary to assess long-term (>48 h) retention and clearance. These considerations underscore the importance of accounting for particle stability, state of particle in biological media, and experimental time scales when extrapolating *in vitro* cellular findings to inhalation risk.

Despite apparent partial dissolution, these data demonstrated that UO_2_ particle toxicity could not be explained by dissolved or colloidal U alone. Instead, toxicity is likely strongly associated with particle internalization, which delivers a highly concentrated U burden directly into cells. This mechanism results in greater cytotoxicity than U_(aq)_ across both macrophages and fibroblasts, supporting the concept that UO_2_ particles act as carriers of localized chemical (and likely radiological) dose at deposition sites. However, the greater toxicity of particulate UO_2_ relative to U_(aq)_ may also reflect a combination of U-specific physicochemical effects and general effects associated with particle internalization, including particle persistence and intracellular burden. The absence of a non-U particulate control in this study limits our ability to fully separate these contributions. Moreover, selection of an appropriate reference particle is itself challenging, as differences in particle morphology, surface chemistry, surface charge, density, agglomeration behavior, and protein corona formation may independently influence cellular uptake and toxicity. Accordingly, the present study should be interpreted as a comparison between particulate and dissolved U under equivalent U exposure conditions, rather than as a full mechanistic separation of U-specific and generic particle-associated effects. Nevertheless, our observation that particulate UO_2_ was more toxic than U_(aq)_ under the tested conditions is consistent with previous studies indicating that particle identity and U physicochemistry influence biological response. Carbon-rich U-bearing particles were substantially more toxic than organosilica and silica control particles in a human lung epithelial cell model,^40^ while different lung epithelial cell models exposed to U_3_O_8_ and UO_3_ displayed varying viability depending on U speciation.^51^ The present study extends these observations through a direct side-by-side comparison of particulate U across multiple lung-relevant cell types.

Macrophages exhibited greater early tolerance to UO_2_ exposure compared with fibroblasts, likely reflecting intrinsic defense mechanisms such as sequestration of particles within lysosomal compartments. However, at higher concentrations and longer exposure times, UO_2_ significantly impaired macrophage viability and function, suggesting that excessive particle burden may compromise clearance capacity. This is consistent with previous publications where, TEM studies of rat alveolar tissues 8 days post-inhalation have shown enlarged macrophages with increased lysosomal granule size following UO_2_ particles exposure, indicative of high particle burden.^58^ The study also reported that the particles have a retention half-life of 160–176 days, reflecting impaired clearance due to macrophage death and UO_2_ re-release. Similar cell-type-dependent sensitivity to U particulates has also been reported in lung epithelial models, where primary cells, derived directly from tissues, have shown greater responses than genetically modified, immortalized cells under comparable conditions,^51^ highlighting the importance of cellular function and particle handling capacity in modulating toxicity. Intracellular imaging further revealed that while some UO_2_ undergoes lysosomal processing, a fraction of nano-sized particles remains structurally intact after 24 h and may persist within, or escape into, the cytoplasm. Such persistent intracellular particles, together with macrophage death and potential extracellular re-release of fragmented material, may promote long-term retention and redistribution of U within lung tissue.

The cell models used here also have limitations when extrapolating these findings to human inhalation exposures. RAW264.7 and Mlg2908 are immortalized murine cell lines, and species-specific differences may influence cellular uptake, inflammatory responses, and clearance. Immortalized lines are also known to differ from primary cells in sensitivity and gene expression, as reported for human lung epithelial models where primary cells showed greater responses than genetically modified immortalized cells under comparable conditions.^51^ These findings should therefore be interpreted as a comparison of particulate and U_(aq)_ behavior under controlled cellular conditions rather than as a direct prediction of human toxicity. Future studies using primary human lung cells, such as human alveolar macrophages and epithelial cells, would improve their relevance to human inhalation exposures. Collectively, these findings highlight the importance of particle form, stability, and cellular handling in determining the biological impact of inhaled U particles, and suggest that particle-associated mechanisms, rather than U_(aq)_ alone, play a central role in driving toxicity, while toxicity for both forms remains dependent on exposure concentration.

## Conclusion

This study has investigated the dissolution behavior, associated cellular response, and intracellular fate of particulate UO_2_ and U_(aq)_ *in vitro* using murine macrophage and lung fibroblast cell models. While UO_2_ is partially dissolved in media and has a minor proportion of colloidal species after 24 h, U_(aq)_ has a higher fraction of colloids under the same conditions. When exposed to the cells, UO_2_ is more toxic across both cell lines and time points at equivalent concentrations. The higher of the two concentrations tested (24 and 60 μg/mL of UO_2_; 100 and 250 μM total U) also triggered a significant inflammatory response as indicated by increased TNF-α secretion after 24 h, but not U_(aq)_ of equivalent concentration. Uptake studies using ultrastructural analysis revealed that most particles reside in compartmentalized vesicles likely to be lysosomes. Nano-sized particles were also observed inside the cytoplasm possibly via lysosomal escape. Further in-depth analysis showed that cytoplasmic particles appear to be mostly intact and have slight structural disruption after 24 h of exposure, implying limited intracellular alteration within the cytoplasm and potential persistence of intracellular UO_2_. Taken together, these results demonstrate that U toxicity *in vitro* is strongly affected by particle form and intracellular fate rather than dissolved or colloidal species alone. This indicates that current risk assessment frameworks should consider the distinct toxicological behavior of particulate U. Future work should employ well-controlled exposure studies using either only nano-sized or only μm-sized particles, to allow better evaluation of size-related effects of U particles on cellular uptake and toxicity. There is also a need to compare dissolution behavior of UO_2_ in cell culture media relative to SLF models, to bridge current discrepancies between the two different model solutions.

## Materials and Methods

### Uranium Sources

Depleted uranium dioxide (UO_2_) (CAS #1344-57-6) was purchased from International Bio-Analytical Industries, Inc. The particles were crushed and sieved to obtain a <5 μm fraction for experiments. As previously reported in a study using the same material (Table S3), SEM analysis confirmed that the particles had an average size of 1.57 ± 1.22 μm and that a large fraction (63%) lies within the size range size range of 1–5 μm.^27^ XRD and TEM also revealed that these grains had a polycrystalline matrix composed of interconnected sub-μm UO_2_ crystallites with diameter of 0.43 ± 0.20 μm.^27^ Uranyl nitrate was obtained by dissolving UO_2_ in 3 M HNO_3_ (analytical grade, Fisher Scientific). For comparison with U_(aq)_, these mass concentrations were converted to U_(aq)_ equivalent molar concentrations based on the U content of UO_2_, assuming complete dissolution of the particles. The corresponding U_(aq)_ equivalent concentrations are provided in Table S4.

All experimental work involving depleted U was conducted in designated radiochemistry laboratories at the University of Helsinki, under appropriate radiation safety licensing and in accordance with institutional radiological safety protocols and Finnish legislation.

### Cell Cultures

RAW264.7 mouse macrophages (TIB-71, ATCC) and Mlg2908 mouse lung fibroblasts (CCL-206, ATCC) were used in the exposure studies. Complete cell culture media was prepared according to supplier recommendation, consisting of Dulbecco’s modified Eagle’ medium (DMEM) and minimum Essential medium Eagle (MEM) for RAW264.7 macrophage and Mlg2908 fibroblast, respectively, with 10% fetal bovine serum (FBS) and 1% penicillin–streptomycin (10,000 U/mL). All were purchased from Life Technologies Gibco.

### Particle Characterization in Complete Cell Culture Media

To assess UO_2_ dissolution and particle behavior in cell culture media, 120 μg/mL of UO_2_ or U_(aq)_ in a 50 mL centrifuge tube were incubated in RAW264.7’s complete cell culture media for 24 h at 37 °C and 95% relative humidity. The solution was then centrifuged at 4000 *g* for 10 min before extracting the supernatant to separate the solids. Size fractionation was then obtained using polytetrafluoroethylene (PTFE) 0.10 μm syringe filters and 3 kDa (∼1.5 nm pore size^59^) cellulose ultracentrifuge filters (Amicon), to obtain the size fraction of >100 nm, 1.5–100 nm, and <1.5 nm. Total U concentrations in the filtrate were then analyzed using microwave plasma atomic emission spectroscopy (Agilent MP-AES 4200). The <0.1 μm fractions were further analyzed by dynamic light scattering (DLS) using a Zetasizer Nano ZS (Malvern). Measurements were performed on 0.1 μm-filtered UO_2_ and U_(aq)_ containing media, with 0.1 μm-filtered and unfiltered complete cell culture media included as controls. Zeta-potential measurements were performed on a Zetasizer Nano ZS (Malvern) to assess protein corona formation on UO_2_ particles. UO_2_ (120 μg/mL) was incubated in complete RAW264.7 culture medium for 24 h at 37 °C with occasional agitation to keep the particles dispersed and permit corona formation. Following incubation, the particles were washed twice with 10 mM TRIS buffer (pH 7.4), with centrifugation (1.41 × 10^4^ g, 5 min) between washes, and resuspended in the same buffer for measurement. TRIS was used as a non-complexing buffer to avoid the uranyl–phosphate surface interactions associated with phosphate-based buffers. As a naked-particle control, UO_2_ (120 μg/mL) was suspended directly in 10 mM TRIS (pH 7.4) and taken through the same washing and measurement steps. Measurements were made in triplicate.

### Thermodynamic Modelling

PhreeqC V3.3.8 ^60^ with the SIT2 database was used to model the dissolution of UO_2_ in DMEM used for RAW264.7. UO_2_ (120 μg/mL) was equilibrated with the media with 5% CO_2_ in air (to mimic the atmosphere of the cell culture incubator) at 37 °C, with the model set to give the maximum total U_(aq)_ concentration, and to define potential secondary U phases as indicated by a positive saturation index. Only the major inorganic salt components of the media were considered, as their concentrations are significantly higher than other components such as amino acids or vitamins. Full media formulations are available in Table S1. The 10% FBS was not explicitly modelled due to its complex and undefined composition, primarily comprising proteins and other organic ligands that are not readily represented in thermodynamic databases. Input files used for the simulations are included in Supporting Information S2.

### Cell Cytotoxicity

RAW264.7 macrophages and Mlg2908 fibroblasts were seeded at a density of 5000 and 10,000 cells per well in a white-walled 96-well plate, respectively, and left to grow overnight. The cells were then treated with 200 μL of complete cell culture media containing UO_2_, or U_(aq)_, with concentrations of 12, 24, 60, and 120 μg/mL for 6, 24, and 48 h, and maintained at 37 °C in a humidified incubator with 5% CO_2_. Complete cell culture media without additives was used as the negative control, while 1% v/v Triton X-100 (Sigma-Aldrich) in complete cell culture media was used as the positive control. Each treatment was performed in triplicate. After exposure, the cells were washed twice with 1× Dulbecco’s phosphate buffered saline (DPBS) at pH 7.4 (Life Technologies Gibco). Cell viability was measured using the CellTiter-glow-Glo assay (Promega). Briefly, 100 μL of 1× HBSS (Hanks Balanced Saline Solution, GE Healthcare Life Sciences) and 100 μL CellTiter-Glo reagent were added to the cells and mixed for 2 min using an orbital shaker (100 rpm) to induce cell lysis. The plate was then left to be incubated at room temperature for 10 min to stabilize any luminescence signal from viable cells. Luminescence readings were then measured using a Synergy H1 Hybrid multimode microplate reader (Agilent BioTek) and were normalized relative to the negative control for comparison. An additional, independent 6 h cytotoxicity experiment was performed using RAW264.7 macrophages exposed to 12, 24, 60, and 120 μg/mL UO_2_, with a cell-free control containing 120 μg/mL UO_2_ included to assess potential direct assay interference. No detectable luminescence signal was observed in the cell-free UO_2_ control (Figure S4), indicating that UO_2_ did not generate a false-positive signal under the assay conditions. However, this control does not fully exclude possible particle-related quenching or enhancement of a true ATP-derived luminescence signal. The CellTiter-Glo results are therefore interpreted as ATP-based viability/metabolic activity measurements, supported by the response patterns observed against changing concentration, time, and cell type.

### Supernatant TNF-α Quantification with Total Protein Normalization

RAW264.7 macrophages and Mlg2908 fibroblasts were seeded at density of 12,500 and 25,000 cells per well in a clear 96-well plate, respectively and left to grow overnight. The cells were then treated with 200 μL of complete cell culture media containing UO_2_ or U_(aq)_, at concentrations of 24 and 60 μg/mL for 24 h at 37 °C, 5% CO_2_ and 95% relative humidity. Cell culture media without additives and 10 ng/mL of lipopolysaccharides (LPS) (Merck) were used as negative and positive controls, respectively. After treatment, both cell culture supernatant and cell lysates were collected for measurement. To measure TNF-α protein concentration in the supernatant, a DY410-05 Mouse TNF-α DuoSet ELISA (R&D Systems) coupled with a DY008B DuoSet ELISA Ancillary Reagent Kit 2 (R&D Systems) were used. TNF-α standards were prepared and measured at concentrations of 31.3, 62.5, 125, 250, 500, 1000, and 2000 pg/mL. The supernatants and standards were added to a 96-well plate coated with the mouse TNF-α capture antibody and prepared according to manufacturer protocol. Subsequently, the TNF-α concentration were measured using the Synergy H1 Hybrid multimode microplate reader set to 450 and 540 nm. To correct for optical imperfections, the finals readings were then obtained by subtracting readings at 540 nm from the readings at 450 nm.

Total protein concentration in each sample was also measured using the Pierce colorimetric bicinchoninic acid (BCA) protein assay (Thermo Fisher Scientific). Briefly, each of the cell lysates collected were lysed with a RIPA lysis and extraction buffer (Merck) with Pierce protease/phosphatase inhibitor (Thermo Fisher Scientific), and the lysed solutions were then diluted 5× with deionized water. Thereafter, 25 μL of each diluted sample was transferred to a new 96-well plate and mixed with 200 μL of BCA working solution. The plates were then wrapped with aluminum foil and allowed to incubate at 37 °C for 30 min. The absorbances were recorded at 562 nm using the Synergy H1 Hybrid multimode microplate reader, and total protein concentration was quantified against a standard curve generated from albumin standards as recommended by manufacturer. The TNF-α protein concentrations of each sample were then normalized with its respective total protein concentration for data analysis.

### Cell Internalization Assay and Total U Measurement

RAW264.7 macrophages and Mlg2908 fibroblasts were seeded on a clear 24-well plate at a density of 10,000 and 20,000 cells per well, respectively. The cultures were allowed to incubate overnight and then replaced with 1 mL of new complete cell culture media containing 12 μg/mL of UO_2_ or U_(aq)_ for 6 and 24 h at 37 °C, 5% CO_2_, and 95% relative humidity. At the designated timepoints, three different fractions were collected: (i) free fraction, (ii) membrane-bound fraction, and (iii) internalized fraction. The free fraction consists of the supernatant (∼1 mL) and 0.5 mL of ice-cold 1× DPBS (pH 7.4) used to wash the cells directly after. Thereafter, the cells were incubated with 0.5 mL of 0.05 M glycine buffer, pH 2.8, prepared from stock glycine (Sigma-Aldrich), for 5 min on ice, twice, and then washed once with ice-cold 1× DPBS (pH 7.4). All these solutions were collected as the membrane-bound fraction. For the internalized fraction, 1 mL of 1 M NaOH was added into the cell pellets for 5 min at room temperature to lyse the cells, followed by 0.5 mL of 1× DPBS (pH 7.4) to suspend any residues in the well.

Samples were then digested with 3 M HNO_3_ (analytical-grade, Fisher Chemical). For each digestion, the fractions were first transferred to an acid-washed 100 mL beaker (5% HNO_3_) and heated to incipient dryness on a hot plate. Then, 20 mL of 3 M HNO_3_ was added and the beaker was covered with a watch glass. Gentle heating was applied to permit a slow boil for 2 h. Once the solution turned clear, the watch glass was removed to evaporate the acid to incipient dryness. Then, 10 mL 5% HNO_3_ was slowly added, washing the sides of the beaker. After further dilution and filtration (0.1 μm PTFE syringe filters), the concentration of U was measured via inductively coupled plasma mass spectrometry (ICP–MS, Agilent 8900). The size fraction distribution was then reported as percentage of the total U concentration as per eq 1.

1 $$measured\;U\;\%=\frac{U\;concentration\;in\;each\;fraction}{sum\;of\;U\;concentration\;in\;\text{all}\;three\;fractions}$$

### Transmission Electron Microscopy Sample Preparation

Cells were first seeded on 13 mm circular glass coverslips in a 12-well plate pre-coated with freshly prepared poly-l-lysine solution (Merck) according to manufacturer protocol. The seeding density was 40,000 cells/mL for RAW264.7 macrophages and 20,000 cells/mL for Mlg2908 fibroblasts, and the cells were allowed to grow overnight. The cells were then treated with 1 mL of complete cell culture media containing UO_2_ or U_(aq)_, at concentrations of 12 μg/mL for 24 h, at 37 °C, 5% CO_2_, and 95% relative humidity. After exposure, cells were immediately fixed with 2% glutaraldehyde (Merck) in 0.1 M sodium cacodylate (NaCac) buffer pH 7.4 (Merck), for 30 min, before being washed twice 0.1 M NaCac for 3 min each. The cells were post-fixed with 1% osmium tetroxide (Merck) in 0.1 M NaCac for 1 h on ice, and then washed twice with 0.1 M NaCac for 3 min each. The cells were then dehydrated using an ethanol series (70%, 96%, and twice with 100%, 1 min each), and then embedded into epoxy resin (hard, TAAB 812, Aldermaston, UK) and at 60 °C for 18 h. Thin sections (60 nm) were cut using an ultramicrotome (Leica EM Ultracut UC7, Leica Mikrosysteme GmbH) and collected on single-slot pioloform-coated copper grids. A section thickness of 60 nm was used for measurements. The sections were then stained with lead citrate and U­(VI)-acetate for imaging.

### TEM Imaging

The sections were first examined by TEM (JEOLJeol JEM-1400) with an electron accelerating voltage of 80 kV. Subsequently, HR-TEM with EDX and high-angle annular dark-field scanning TEM (HAADF–STEM) were performed using a JEOL JEM-ARM200F with an acceleration voltage of 200 kV to further examine chemical and mineralogical variations at the sub-μm scale within individual particles. JEOL Analysis Station software was used for the control of STEM–EDX mapping. A drift-correction mode was used during acquisition of elemental maps to reduce effects of sample drift. The STEM probe size was 0.13 nm, generating at a current of 140 pA when a condenser lens aperture of 40 μm was inserted. The HAADF detector was set to collect signals from range of ∼97 mrad (inner) to 256 mrad (outer).

## Supplementary Material



## Notes

Chemical composition for cell culture media (Table S1); UO_2_ dissolution and stability in DMEM, PhreeqC input and selected output files of UO_2_ and U_(aq)_ (Supporting Information 2.1–2.3); TEM image and HAADF–STEM analysis of an ultrathin section (∼60 nm) of RAW264.7 macrophage exposed to 12 μg/mL of UO_2_ (Figure S1); and TEM image and HAADF–STEM analysis of an ultrathin section (∼60 nm) of Mlg2908 macrophage exposed to 12 μg/mL of UO_2_ (Figure S2). Additional particle characterization data (Table S4), additional details on DLS measurement (Supporting Information S4.2), additional DLS data (Figure S3). U concentration data for experiments (Table S4). Additional control data for the CellTiter-Glo assay (Figure S4) (PDF)
