# Supplementary material for: Toxicity, Cellular Uptake, and Intracellular Fate of Particulate and Dissolved Uranium in Murine Lung-Relevant Cell Models
Source: ACS Omega. 2026 Sep 25;11(39):59601–12. doi: 10.1021/acsomega.6c06055 (PMC13647355; doi:10.1021/acsomega.6c06055)
Supplement: Supplementary file 1 [file ao6c06055_si_001.pdf]

Supporting information (SI) for:

Toxicity, cellular uptake, and intracellular fate of particulate and dissolved uranium in murine lung-relevant cell models.

You Cheng Khng[1,2], Gianni F. Vettese[1], Satoshi Utsunomiya[3], Anna M. Psyrillou[1], Ole Christian Lind[4] ,Per Malmberg[5], Mirkka Sarparanta[1], Gareth T.W. Law[1*]

[1]Radiochemistry Unit, Department of Chemistry, The University of Helsinki, Helsinki 00560, Finland

[2]Singapore Nuclear Research and Safety Institute, National University of Singapore, Singapore 118415, Singapore

[3]Department of Geosciences, National Taiwan University, No. 1, Sec. 4, Roosevelt Road, Taipei 106, Taiwan

[4]Faculty of Environmental Sciences and Natural Resource Management, Norwegian University of Life Sciences, P.O. Box 5003, NO-1432 Ås, Norway

[5]Department of Chemistry and Chemical, Engineering, Chalmers University of Technology, 412 96, Göteborg, Sweden

*Corresponding author: Gareth.law@helsinki.fi

The SI contains 10 pages, 4 Figures, and 3 Tables, presenting further information on methods, additional data, and additional interpretation.

Table of Contents

S1. Chemical composition for cell culture media

S2. UO_2 Dissolution and stability in DMEM

S2.1. PhreeqC input

S2.2. Selected output files for UO_2 in DMEM

S2.3. Selected output files for $U_{(aq)}$ in DMEM

S3. Addition TEM image analysis

S3.1. RAW264.7 macrophage exposed to 12 µg/mL of UO_2

S3.2. Mlg2908 fibroblast exposed to 12 µg/mL of UO_2

S4. Additional information on particle characterization in complete cell culture media

S4.1. Particle characterization of UO_2 starting material

S4.2. Additional details on DLS measurement

S5. Conversion of U concentration from µg/mL to µM U equivalent

S6. Cell-free control setup to check for assay interference

S1. Chemical composition for cell culture media

Table S1. DMEM and MEM media composition according to manufacturer (Life Technologies Gibco); simplified DMEM was synthesized based on dominant inorganic composition.

	Simplified DMEM (mg/L)	DMEM (mg/L)	MEM (mg/L)
**Inorganic salts**			
Calcium chloride (anhyd.)		200	200
Ferric nitrate		0.1	54.9
Magnesium sulfate (anhyd.)		97.67	97.67
Potassium chloride		400	400
Sodium bicarbonate	3700	3700	2200
Sodium chloride	6400	6400	6800
Sodium phosphate monobasic		125	140
**Amino acids**			
Glycine		30	
L-arginine hydrochloride		84	126
L-Cystine-2HCl		63	31
L-Glutamine		584	292
L-Histidine hydrochloride-H2O		42	42
L-Isoleucine		105	52
L-Leucine		105	52
L-Lysine hydrochloride		146	73
L-Methionine		30	15
L-Phenylalanine		66	32
L-Serine		42	
L-Threonine		95	48
L-Tryptophan		16	10
L-Tyrosine disodium salt dihydrate		104	52
L-Valine		94	46
**Vitamins**			
Choline chloride		4	1
D-Calcium pantothenate		4	1
Folic acid		4	1
i-Inositol		7.2	2
Niacinamide		4	1
Pyridoxine hydrochloride		4	
Riboflavin		0.4	0.1
Thiamine hydrochloride		4	1
**Other components**			
D-glucose (Dextrose)		4500	1000
Phenol Red		15	10
Sodium Pyruvate		110.0	110.0
**pH**	7.4**	7.4*	7.4*

*Adjusted using HCL only

**Only when media is incubated with 5% CO_2.

S2. UO_2 Dissolution and stability in DMEM

S2.1. PhreeqC input

```
DATABASE c:\phreeqc\database\Sit2.dat #
TITLE Uranium dioxide dissolution in cell culture media
SOLUTION_MASTER_SPECIES
	# redox-uncoupled gases
	Ntg		Ntg		0	Ntg	28.0134 # N2 gas
	Oxg		Oxg		0	Oxg	32.0 #O2 inert gas
SOLUTION_SPECIES
	H2O + 0.01e- = H2O-0.01
		log_k -9.0

	Ntg = Ntg # N2
		log_k 0
		delta_h   0.000
		# -dw      1.96e-9
		# -Vm 7 # Pray et al., 1952, IEC 44. 1146
	Oxg = Oxg #O2
		log_k 0
		delta_h 0
PHASES
H-Autunite
	H2(UO2)2(PO4)2 = 2H+ + 2UO2+2 + 2PO4-3
	log_k   -47.931
	delta_h -3.6    kcal
Na-Autunite
	Na2(UO2)2(PO4)2 = 2Na+ + 2UO2+2 + 2PO4-3
	log_k   -47.409
	delta_h -0.46   kcal
K-Autunite
	K2(UO2)2(PO4)2 = 2K+ + 2UO2+2 + 2PO4-3
	log_k   -48.244
	delta_h 5.86    kcal
Autunite
	Ca(UO2)2(PO4)2 = 2UO2+2 + Ca+2 + 2PO4-3
		log_k   -43.927
		delta_h -14.34  kcal
Ntg(g)
	Ntg = Ntg
	-analytic -58.453 1.81800e-3  3199  17.909 -27460
	T_c  126.2 ; -P_c   33.50 ; -Omega 0.039
Oxg(g)
	Oxg = Oxg
	-analytic -7.5001 7.8981e-3 0.0 0.0 2.0027e5
	-T_c  154.6 ; -P_c   49.80 ; -Omega 0.021
O2(g)
	O2 = O2
	-log_k   -2.8983
	-analytic -7.5001 7.8981e-3 0.0 0.0 2.0027e5
	-T_c  154.6; -P_c   49.80; -Omega 0.021
CO2(g)
	CO2 = CO2
	-log_k    -1.468
	-delta_h -4.776 kcal
	-analytic   10.5624  -2.3547e-2  -3972.8  0  5.8746e5  1.9194e-5
	-T_c  304.2 # critical T, K
```

```
            -P_c   72.86 # critical P, atm
            -Omega 0.225 # acentric factor

SOLUTION 1 DMEM
            units               mmol/L
            density   1.0 #assume 1
            temp                37.0
            redox               pe #O(0)/O(-2)
            pH                  7.4
            pe                              12 O2(g) -0.68
            Na                  154.4726
            Cl                  113.1183
            P                   0.916197
            Ca                  1.802127
            C                   44.04238
            K                   5.365526
            S                   0.811347
            Mg                  0.811347
END

USE Solution 1
EQUILIBRIUM_PHASES #Adding 4.44e-4 (120 ug/mL) of UO2 into DMEM
            Uraninite 0 4.44e-4
            CO2(g)     -1.301  #Log 5% CO2

USER_PRINT
-start
10 t = SYS("phases", count , name$ , type$ , sis )
20 FOR i = 1 to count
30  IF (sis(i) < 0) THEN GOTO 50
40  PRINT PAD(name$(i),10), STR_F$(sis(i),6,2)
50 NEXT i
-end
END

USE Solution 1
REACTION 1 #Add uranyl nitrate into DMEM
            UO2(NO3)          1
            4.44e-4 moles in 1 steps
EQUILIBRIUM_PHASES
            Co2(g)  -1.301

USER_PRINT
-start
10 t = SYS("phases", count , name$ , type$ , sis )
20 FOR i = 1 to count
30  IF (sis(i) < 0) THEN GOTO 50
40  PRINT PAD(name$(i),10), STR_F$(sis(i),6,2)
50 NEXT i
-end
END
```

S2.2. Selected output files for UO_2 in DMEM

--Phase assemblage---

				Moles in assemblage		
Phase	SI	log IAP	log K(T, P)	Initial	Final	Delta
Uraninite	-24.67	-30.05	-5.38	4.44E-04	0	-4.44E-04

---------------------------Description of solution---------------------------

pH = 7.633 Charge balance

---------------------------Distribution of species---------------------------

Species	Molarity	Activity	Log Molarity	Log Activity	Log Gamma	mole V cm³/mol
U(4)	2.454e-39					
U(6)	4.44E-04					
CaUO2(CO3)3-2	1.72E-04	5.11E-05	-3.763	-4.292	-0.529	0
UO2(CO3)3-4	1.41E-04	1.11E-06	-3.850	-5.953	-2.104	0
Ca2UO2(CO3)3	1.15E-04	1.15E-04	-3.938	-3.938	0	0
MgUO2(CO3)3-2	1.40e-05	4.15e-06	-4.854	-5.382	-0.529	0

S2.3. Selected output files for $U_{(aq)}$ in DMEM

---------------------------Description of solution---------------------------

pH = 7.629 Charge balance

---------------------------Distribution of species---------------------------

Species	Molarity	Activity	Log Molarity	Log Activity	Log Gamma	mole V cm³/mol
U(4)	2.846e-40					
U(6)	4.44E-04					
CaUO2(CO3)3-2	1.72E-04	5.11E-05	-3.763	-4.292	-0.529	0
UO2(CO3)3-4	1.41E-04	1.11E-06	-3.850	-5.953	-2.104	0
Ca2UO2(CO3)3	1.16E-04	1.16E-04	-3.938	-3.938	0	0
MgUO2(CO3)3-2	1.40e-05	4.15e-06	-4.854	-5.382	-0.529	0

S3. Additional TEM data

S3.1. RAW264.7 macrophage exposed to 12 µg/mL of UO_2

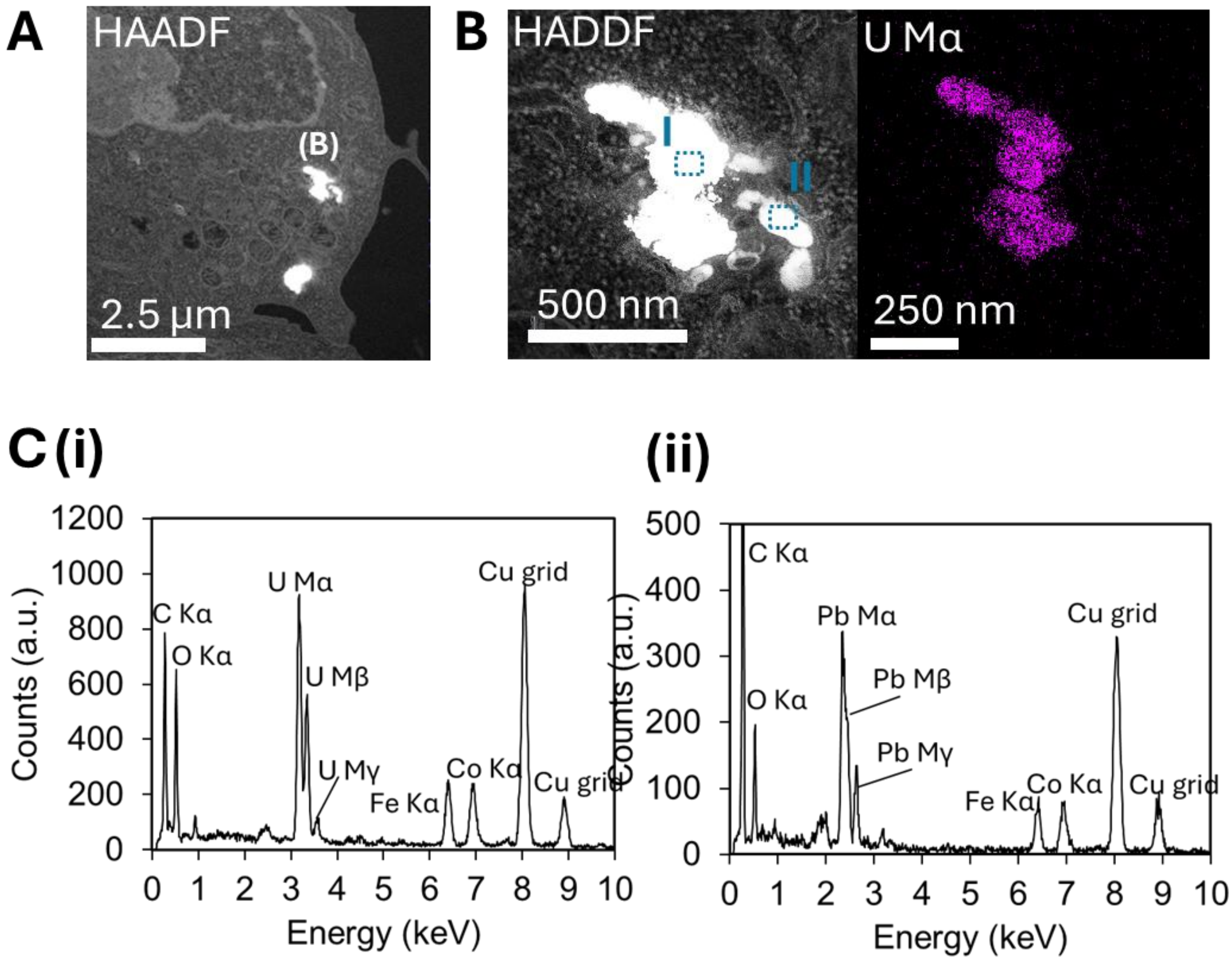

Figure S1. HAADF-STEM analysis of an ultrathin section (~60 nm) of RAW264.7 reacted to 12 µg/mL of UO_2 for 24 h. (A) HAADF image UO_2 particles. The particle of interest is denoted as (B). (B) Zoomed in HAADF image and EDX mapping of U (purple) of UO_2 particle marked in (A). (C) STEM-EDX spectrum of regions as labelled in (B). Region I refers to the core of the particle UO_2 particle and region II refers to an adjacent Pb precipitate.

S3.2. Mlg2908 fibroblast exposed to 12 µg/mL of UO_2

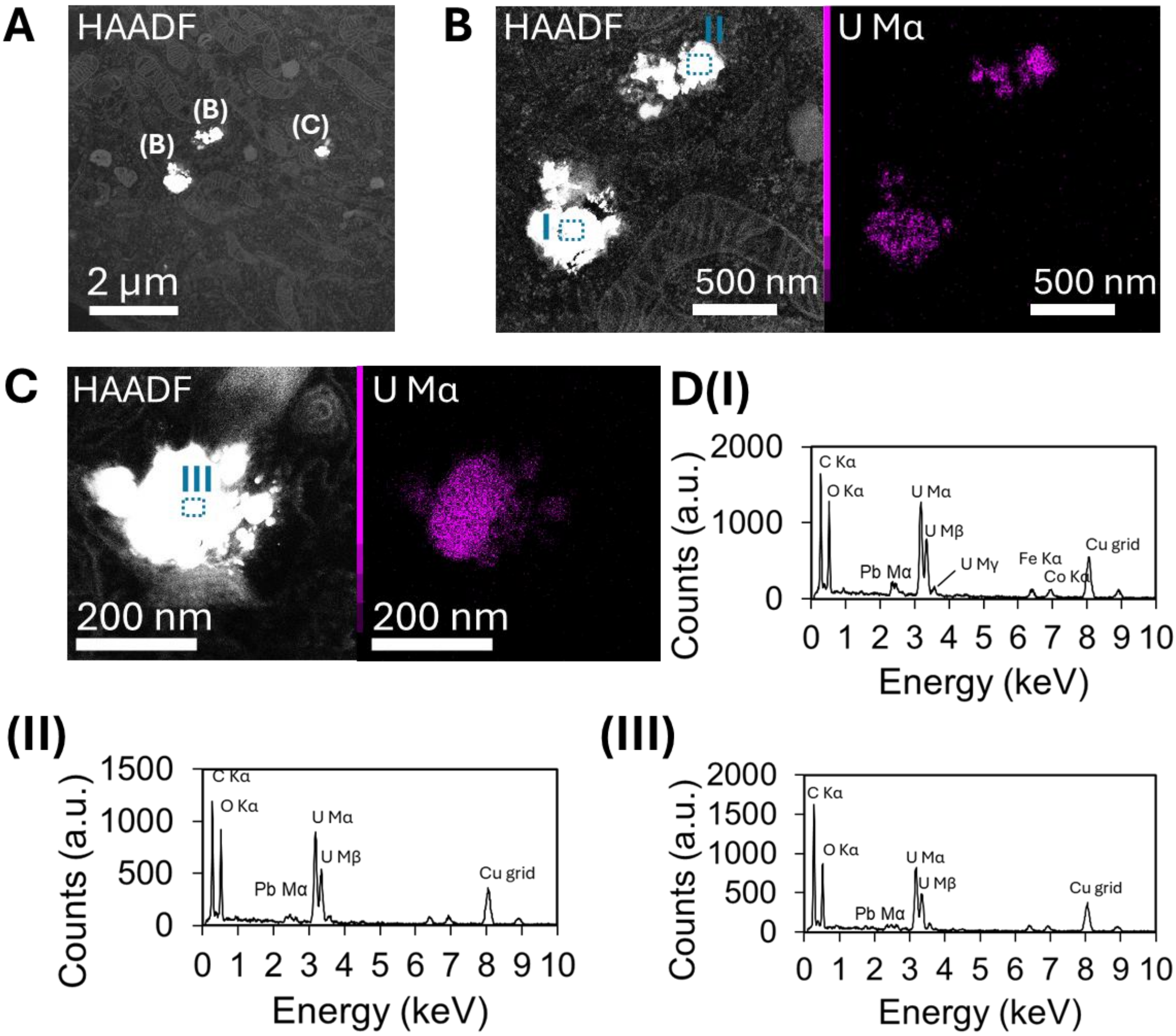

Figure S2. HAADF-STEM analysis of an ultrathin section (~60 nm) of Mlg2908 reacted to 12 µg/mL of UO_2 for 24 h. (A) HAADF image. Particles of interest are denoted as (B) and (C). (B) Zoomed in HAADF image and EDX mapping of U (purple) of two UO_2 particles marked in (A). (C) Zoomed in HAADF image and EDX mapping of U (purple) of another UO_2 particle marked in (A). (D) STEM-EDX spectrum of particles I, II and III, as labelled in (B) and (C).

S4. Additional information on particle characterization in complete cell culture media

S4.1. Particle characterization of UO_2 starting material

Table S2. Summary of the characteristics of the UO_2 particles used in this study, as previously reported.[1]

Characteristic	Method	Reported value
Particle size	SEM with ImageJ	1.57 ± 1.22 µm
Particle-size fraction	SEM with ImageJ	63% within 1–5 µm
Particle structure	TEM	Polycrystalline matrix composed of interconnected sub-µm crystallites
Crystallite size	TEM	0.43 ± 0.20 µm
Crystalline phase	XRD	UO_2

S4.2. Additional details on DLS measurement

Figure S3 shows the intensity-weighted size distribution of UO_2-in-media (<0.1 µm fraction), $U_{(aq)}$-in-media (<0.1 µm fraction), media-only control (<0.1 µm fraction), and media-only control (unfiltered). The colloid populations detected in the UO_2 and $U_{(aq)}$ suspensions closely match those of the media-only control (<0.1 µm fraction), indicating that these colloids originate from the media rather than from added U. As DLS is intensity-weighted and over-represents larger particles, and complete media contains abundant serum-derived colloids, individual populations cannot be unambiguously assigned to U; the data are therefore interpreted as showing no resolvable U-specific colloidal population above the media background.

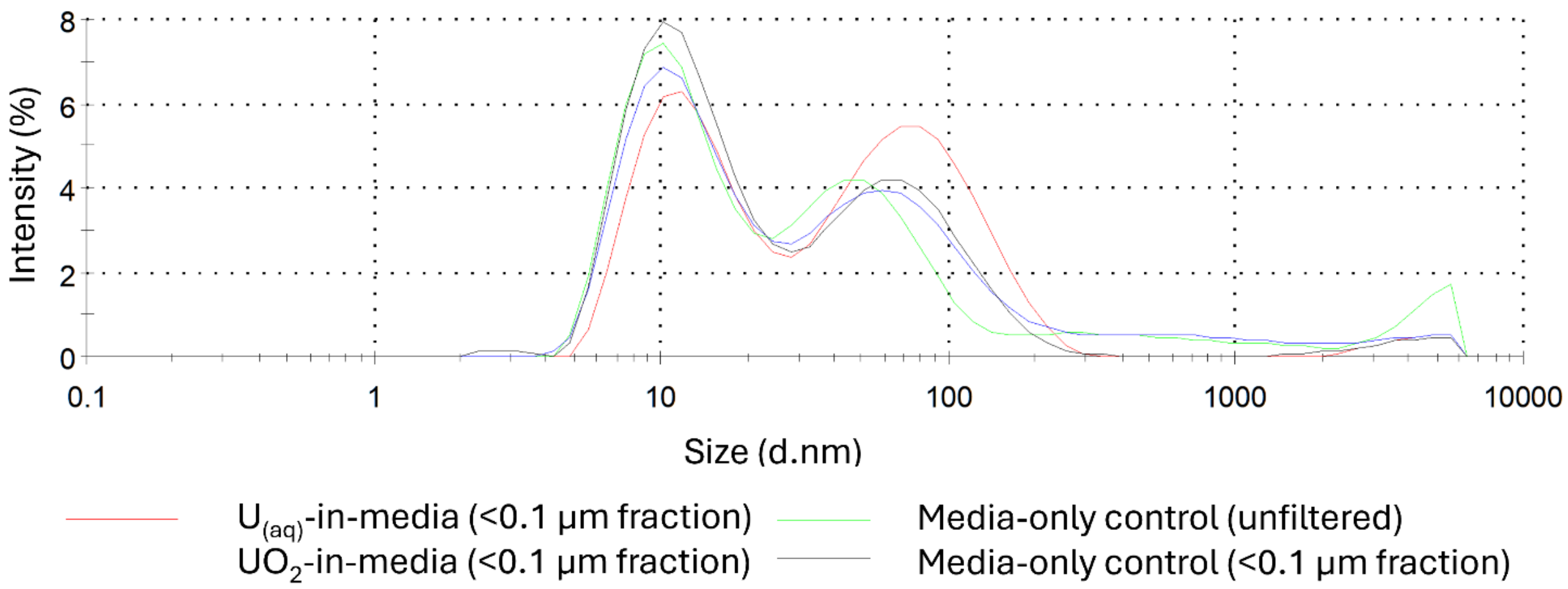

Figure S3. DLS intensity-weighted size distributions of the <0.1 µm (colloidal) fraction of cell culture media suspensions following incubation of 120 µg/mL UO_2 (blue) or $U_{(aq)}$ (red) in complete cell culture medium. Media-only controls with (black) and without (green) 0.1 µm filtration were also measured.

S5. Conversion of U concentration from µg/mL to µM U equivalent

Table S3. Conversion of UO_2 mass concentrations (µg/mL) to equivalent molar U concentrations (µM U), assuming complete dissolution of UO_2.

Mass concentration (µg/mL)	Equivalent molar U concentration (µM)
12	50
24	100
60	250
120	500

S6. Cell-free control experiment to check for assay interference

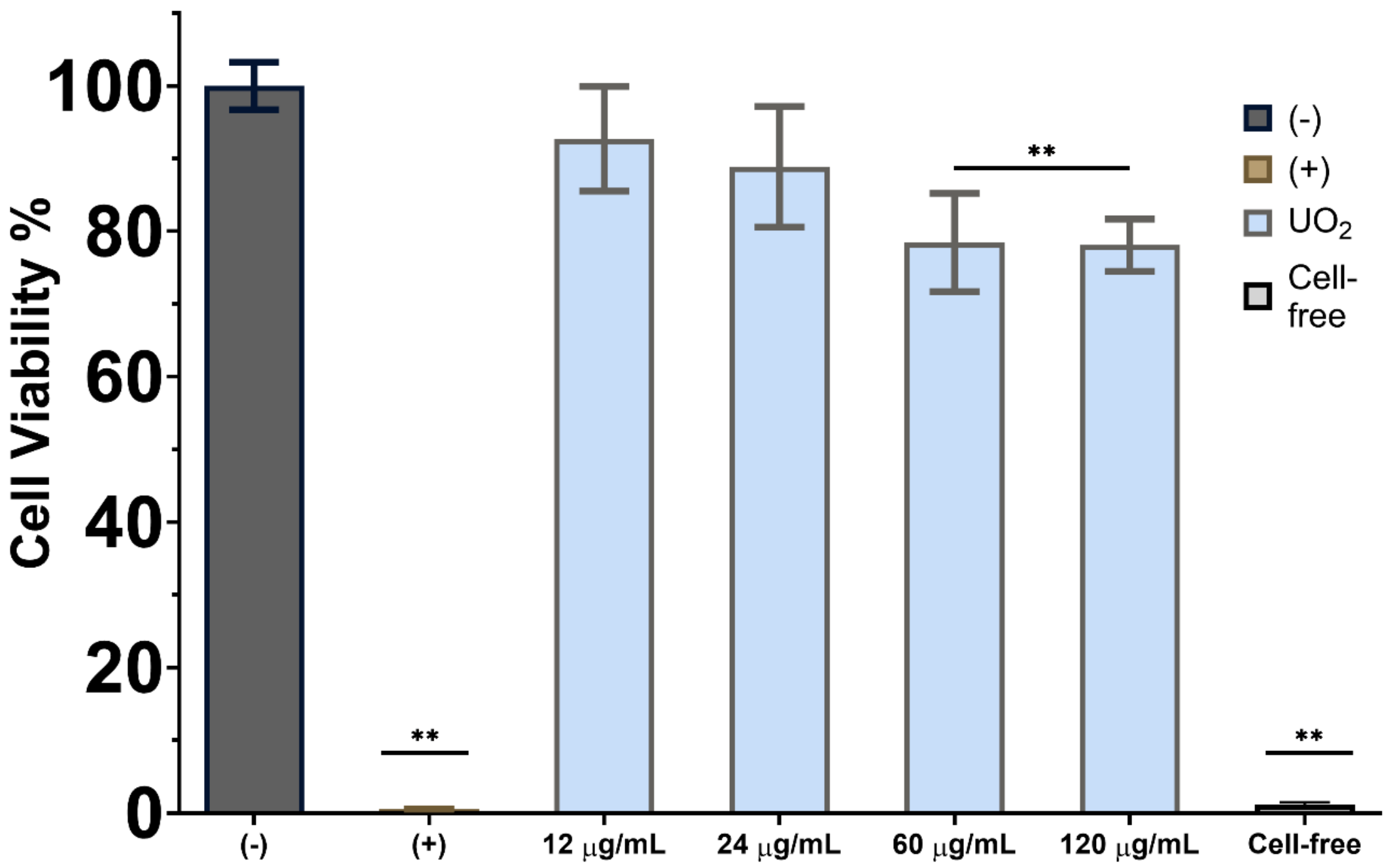

Figure S4. Additional independent cytotoxicity study of RAW264.7 macrophage following exposure to cell culture medium (negative control), 1% Triton-X (positive control), and UO_2 at concentrations of 12, 24, 60, and 120 µg/mL for 6 h. A cell-free control containing 120 µg/mL UO_2 is included to assess potential luminescence interference with CellTiter-Glo assay. The cell viability % was obtained by normalizing against negative control. Data are from triplicates (n=3). The statistical significance was analyzed using one-way ANOVA and post-hoc Tukey test, where the significance was set at *p<0.05 and **p<0.01.

References

(1) Khng, Y. C.; Vettese, G. F.; Utsunomiya, S.; Ang, J. W. L.; Walker, J. M.; Parker, J.; Neill, T.; Morris, K.; Abrahamsen–Mills, L.; Sarparanta, M.; et al. Chemical alteration of UO_2 micro-particles in model lung systems. *J. Hazard. Mater.* **2025**, *497*, 139670.
